# The feasibility of implementing a cultural mentoring program alongside pain management and physical rehabilitation for chronic musculoskeletal conditions: results of a controlled before-and-after pilot study

**DOI:** 10.1186/s12891-022-06122-x

**Published:** 2023-01-19

**Authors:** Bernadette Brady, Balwinder Sidhu, Matthew Jennings, Robert Boland, Geraldine Hassett, Lucy Chipchase, Clarice Tang, Sylvia Yaacoub, Natalie Pavlovic, Samia Sayad, Toni Andary, Shaniya Ogul, Justine Naylor

**Affiliations:** 1 Liverpool Hospital, South Western Sydney Local Health District, Locked Bag 7103 Liverpool, BC, Sydney, NSW 1871 Australia; 2 School of Health Sciences, Western Sydney University, Locked Bag 1797, Penrith, NSW 2751 Australia; 3 Sydney School of Health Sciences, Faculty of Medicine and Health, The University of Sydney, Sydney, NSW 2006 Australia; 4 Multicultural Health Unit, South Western Sydney Local Health District, 5/39 Stanley Street, Bankstown, NSW 2200 Australia; 5 Fairfield Hospital, South Western Sydney Local Health District, PO Box 5, Fairfield, Sydney, NSW 1851 Australia; 6 Caring Futures Institute, College of Nursing and Health Sciences, Flinders University, Sturt Rd, Bedford Park, SA 5042 Australia; 7 South Western Sydney Local Health District, Locked Bag 7103, Liverpool BC, Sydney, NSW Australia; 8 SWS Clinical School UNSW, Locked Bag 7103, Liverpool BC, Sydney, NSW 1871 Australia; 9 Ingham Institute Applied Medical Research, 1 Campbell St, Liverpool, Liverpool, NSW 2170 Australia

**Keywords:** Culturally and linguistically diverse, Cultural mentor, Natural helper, Patient activation, Feasibility

## Abstract

**Background:**

Culturally diverse communities face barriers managing chronic musculoskeletal pain conditions including navigation challenges, sub-optimal healthcare provider engagement and difficulty adopting self-management behaviours.

**Objectives:**

To explore the feasibility and trends of effectiveness of implementing a cultural mentoring program alongside clinical service delivery.

**Methods:**

This quasi-experimental controlled before-and-after multiple case study was conducted in three hospital-based services that provide treatment for patients with musculoskeletal pain. Two prospective cohorts, a pre-implementation and a post-implementation cohort, of adults with chronic musculoskeletal pain who attended during the 6-month recruitment phase, were eligible if they self-identified with one of the cultures prioritised for mentoring by the clinic. The pre-implementation cohort received routine care for up to 3-months, while the post-implementation cohort received up to 3-months of cultural mentoring integrated into routine care (3 to 10 sessions), provided by a consumer (*n* = 6) with lived experience. Feasibility measures (recruitment and completion rates, attendance, satisfaction), and trends of effectiveness (Patient Activation Measure and Health Literacy Questionnaire items one and six) were collated over 3-months for both cohorts. Outcomes were presented descriptively and analysed using Mann-Whitney U-tests for between-group comparisons. Translation and transcription of post-treatment semi-structured interviews allowed both cohorts’ perspectives of treatment to be analysed using a Rapid Assessment Process.

**Results:**

The cultural mentor program was feasible to implement in clinical services with comparable recruitment rates (66% pre-implementation; 61% post-implementation), adequate treatment attendance (75% pre-implementation; 89% post-implementation), high treatment satisfaction (97% pre-implementation; 96% post-implementation), and minimal participant drop-out (< 5%). Compared to routine care (*n* = 71), patients receiving mentoring (*n* = 55) achieved significantly higher Patient Activation Measure scores (median change 0 vs 10.3 points, *p < 0.01*) at 3-months, while Health Literacy Questionnaire items did not change for either cohort over time. Three themes underpinned participant experiences and acceptability of the mentoring intervention: ‘expectational priming’, ‘lived expertise’ and ‘collectivist orientation’ to understand shared participant experiences and explore the potential differential effect of the mentoring intervention.

**Conclusion:**

Participant experiences and observations of improved patient activation provide support for the acceptability of the mentoring intervention integrated into routine care. These results support the feasibility of conducting a definitive trial, while also exploring issues of scalability and sustainability.

**Supplementary Information:**

The online version contains supplementary material available at 10.1186/s12891-022-06122-x.

## Background

Musculoskeletal pain is a leading cause of disability globally [1] and is recognized to burden culturally and linguistically diverse (CALD) communities more than the others [2]. People from CALD communities are observed to have a higher prevalence of persistent pain, more widespread pain, and are more likely to experience inadequate pain control [2–4]. Such disparities are not driven by isolated factors, but rather the intersection of multiple potential vulnerabilities [5–7]. Challenging migration circumstances, family and community dispersion and the experience of trauma are known to amplify pain-related distress [8, 9]. Socio-economic circumstances may constrain access to preventative and rehabilitative healthcare, while language and health literacy challenges contribute to difficulties navigating complex health systems [10, 11]. At the interface with the health system, CALD patients experience additional challenges including, potentially discordant perspectives of health from those held by their healthcare providers [12, 13] and encounters of implicit and explicit bias [14, 15]. Such challenges may account for low rates of engagement with evidence-based pain management amongst some CALD communities and contribute to poorer outcomes [16–19]. Combined, these factors highlight a critical need for targeted approaches to minimise the inequities observed for people from CALD communities living with pain conditions.

The practice of cultural responsiveness in pain management is considered important for responding to the disparities in outcomes experienced by CALD communities [2, 18]. Defined as ‘*health care services that are respectful of, and relevant to, the health beliefs, health practices, culture and linguistic needs of diverse consumer/patient populations*’ [20], cultural responsiveness encompasses both the knowledge and capacity to respond to the needs of diverse communities. Despite arguments for the importance of culturally responsive healthcare practices in Australia and internationally, there is limited research to guide healthcare providers to operate effectively in culturally responsive ways [21, 22]. While cultural competence training programs have demonstrated efficacy for increasing healthcare provider awareness of the needs of diverse communities, there is little evidence that they improve patient outcomes [23]. In a pain management context, treatment programs targeted to a specific language or ethnocultural community provide preliminary evidence of improved patient engagement and/or pain outcomes compared to usual care [19, 24]. However, they risk promoting generic treatment models that fail to address variations between different ethnocultural communities [25] and are not scalable for the breath of cultures and diversity of communities in multicultural societies such as Australia [7]. Rather, there is a need for healthcare providers and healthcare settings to integrate sociocultural context in the clinical reasoning and treatment planning processes for individual patients and not just those from specific cultural backgrounds [22].

One potential strategy for supporting healthcare providers to integrate sociocultural context in their management of patients from CALD backgrounds is through consumer partnership models. There is extensive literature documenting the value of consumer or peer support for people participating in chronic disease self-management or health promotion programs [26, 27]. Specifically, the integration of peer support or mentor programs is reported to improve patient understanding of health problems, the adoption of active coping strategies and navigation of complex health systems [27, 28]. For ethnocultural minority communities, mentorship or navigation provided by someone who identifies with a similar cultural background and/or language has been documented to contribute to social (financial stresses, community integration) and health-related outcomes (e.g. lifestyle change, disease control and cognitive outcomes) [29, 30]. Despite these promising findings, there is limited evidence exploring the integration of consumers or peers in clinical settings to support people with musculoskeletal pain conditions, especially those who identify with a CALD community [31, 32]. Further research is warranted to investigate the unrealized healthcare potential of these valuable community assets.

## Methods

### Aims/objectives

This study explored the feasibility of embedding a consumer mentor initiative in three clinical services for patients with chronic musculoskeletal pain conditions. We adopted the term ‘*natural helper’, as previously described by Dennis* et al [33], to describe our prospective mentors as someone intrinsically motivated to help others in everyday life. For feasibility aims, the research question asked if it was feasible to implement and evaluate a ‘natural helper’ mentoring initiative alongside routine care in different hospital-based clinical services for patients with musculoskeletal pain conditions. Feasibility was described in terms of participant recruitment, intervention acceptability, and study retention. For trends of clinical effectiveness, outcomes were exploratory and sought to document the influence of a ‘natural helper’ mentoring initiative alongside routine care on patient activation, engagement and satisfaction with care.

### Design

**Fig. 1:**
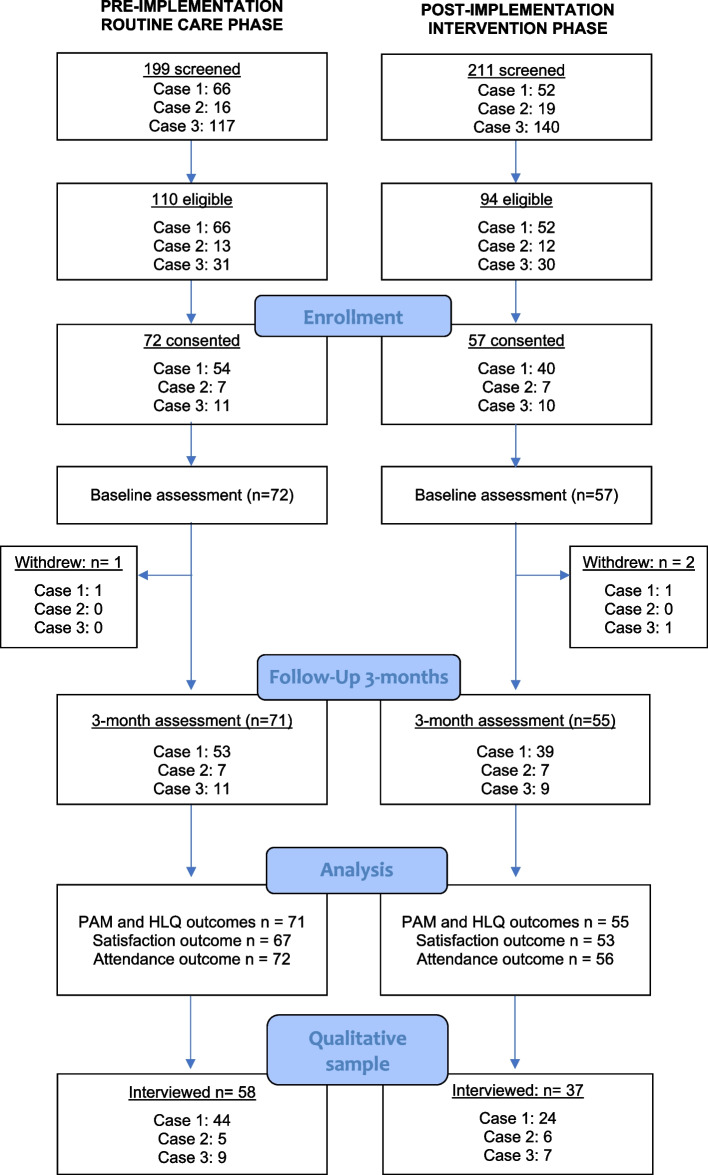
Flow diagram of study processes

This was a prospective quasi-experimental controlled before-and-after multiple case study conducted in three hospital-based clinics across two metropolitan public hospitals between December 2019 and July 2021. For this multiple-case design research, the unit of analysis was the *‘natural helper’* program, the cases were the clinics in which the initiative was embedded and the context was the public hospital and its geographical location. An evaluative multiple case design using mixed methods data sources was selected to allow the research team to explore issues related to implementation across different settings and contexts and provide insight into factors associated with feasibility, acceptability and outcome trends that could guide future implementation research [34]. Figure 1 outlines the participant flow and study processes. The three cases included a tertiary pain clinic (case one), a musculoskeletal physiotherapy outpatient service (case two) and an orthopaedic hip and knee service (case three) for people with end-stage osteoarthritis awaiting joint replacement surgery. This study was approved by the South-Western Sydney Local Health District Human Research Ethics Committee (2019/ETH12816).

### Participants and recruitment

Across the three cases, potential participants were identified from consecutive new patients meeting the eligibility criteria and attending one of the services during either a pre-implementation (routine care) or post-implementation (intervention) recruitment phase. Initially, a three-month recruitment period was expected to be sufficient to achieve key aims. However, changes imposed by the Covid-19 pandemic resulted in fewer patients attending services and as such the recruitment period was extended to 6-months. Prospective participants were informed about the study by clinical staff and those interested in participating were provided with further information and screened for eligibility by a research officer at their clinic appointment or via telephone. An interpreter or bilingual member of the research team was present for study discussion and consenting for all prospective participants who spoke Arabic, Assyrian or Vietnamese. Written informed consent was obtained for all participants, using translated documents, where relevant.

Prospective participants were eligible if they: 1) were aged ≥18 years, 2) were diagnosed with a musculoskeletal pain condition by their treating healthcare provider, 3) were anticipated to attend treatment with the service at least three times during the 3-month intervention or routine care period, 4) self-identified with the target culture specified by the clinic they were attending based on language, ethnocultural or other cultural identification, 5) consented to participate in the research evaluation and/or interviews. The target cultures identified for each case varied according to the geographical location and prominence of specific CALD communities. Assyrian and Arabic-speaking communities were specifically targeted by cases two and three, while case one included a broader target cohort including consecutive patients of any cultural identification who spoke English, Arabic or Vietnamese. Prospective participants were excluded if they did not have a musculoskeletal pain diagnosis, were not expected to receive the minimum therapy dose and/or did not speak or read one of the languages targeted for mentoring sufficient to give written informed consent (Arabic, Assyrian, Vietnamese or English).

### Pre-implementation or routine care phase

Consenting participants recruited during this phase attended routine care according to the specific clinic/case they were enrolled. For case one, this was a 6–8 week language-specific pain management program, for case two a six-week physiotherapy education and exercise program and for case three a combined pre-operative preparedness workup and early post-operative joint replacement rehabilitation. All interventions were conducted in the preferred language of participants, using an accredited health language interpreter and delivered or overseen by healthcare providers appointed to Senior positions in their respective professions (Nursing, Physiotherapy and Psychology). Within each case, treating healthcare providers used their clinical judgement to tailor the interventions/programs to each patient, according to principles of patient-centered care.

### Post-implementation or intervention phase

Consenting participants recruited to this phase attended the same structure of treatment as routine care, with the addition of a ‘natural helper’ mentor. Within each service, one mentor who had a lived experience of musculoskeletal pain relevant to the case (chronic musculoskeletal pain for cases one and two, and end-stage hip or knee osteoarthritis for case three) and identified with the target community was embedded to provide mentoring that complimented routine care. All mentors had completed a co-designed mentor training program, undergone mandatory human resource onboarding requirements and signed a code of conduct. Importantly, mentors were not designed to be paraprofessionals, but past consumers of the health service with lived experience of the condition and community for which they were mentoring [33]. Characteristics of the mentoring program varied for each case, designed to complement routine care and ensure healthcare providers were able to oversee and supervise the activities of mentors. Each mentor was debriefed by a healthcare provider following each mentoring session to ensure clinical governance and fidelity to the agreed aim/content. Table 1 summarises the specific aims and format of Natural Helper mentoring provided in each clinic and the logic model outlining the proposed outcomes associated with the pilot intervention.

**Table 1:** Logic Model describing the aims, format and intended outcomes of the mentoring intervention

Case	Target	Mentoring Intervention Characteristics	Outputs	Impact
1: Tertiary pain clinic	To facilitate patient activation and engagement with an 8-week low-intensity multidisciplinary pain management program	**What**: Emotional, informational and appraisal support^33^ to assist people with chronic pain to participate in a pain management program and adopt self-management behaviours. **Where**: Virtual* mentor and patients **Who**: Arabic (*n* = 1), Vietnamese (*n* = 1), English speaking (*n* = 2) natural helpers with chronic musculoskeletal pain who previously attended a pain management program. **How**: Natural Helpers participated in the pain management program, shared their lived experiences with the group and debriefed each participant (minimum of 15 minutes) individually during each session (6–8 sessions).	**FEASIBILITY MEASURES** Number of participants recruited from each setting. Demographics of people recruited and consenting to mentoring. Dose and format of mentoring preferred and/or adopted by each setting Attendance at scheduled appointments Satisfaction with treatment (CSQ-8 measure) Patient participation in mentoring sessions Completion and return of outcome measures Perceptions of and satisfaction with study processes (qualitative) **EFFECTIVENESS MEASURES** Clinical outcome trends for: - Change in ‘activation’ (PAM: primary measure) - Change in engagement (HLQ items 1 and 6) (secondary measure)	Design of future cluster RCT Choice of outcome measures Define core elements of the mentoring intervention Identify future implementation strategies
2: Physiotherapy Musculoskeletal Outpatient Service	To facilitate patient activation and engagement with a 6-week physiotherapy education and exercise program.	**What**: Emotional, informational and appraisal support^33^ to assist people with musculoskeletal pain to participate in exercise therapy. **Where**: Virtual* mentor with patients in the clinic. **Who**: Assyrian and Arabic speaking (n = 1) natural helper with chronic musculoskeletal pain (knees and low back) who previously attended a physiotherapy exercise program and continued with self-management. **How**: The Natural Helper virtually joined the program, participated in education sessions and shared their lived experiences followed by an individual debrief with each participant (minimum of 15 minutes) during each session (6 sessions).		
3: Orthopaedic Hip and Knee Service	To facilitate patient activation, preparedness and engagement with hip or knee arthroplasty rehabilitation over a 12-week period.	**What**: Emotional, informational and appraisal support^33^ to assist people with end-stage osteoarthritis of the hip and knee to prepare for their surgery and engage in early post-operative rehabilitation. **Where**: Hybrid: Virtual* mentor with participants in clinic and phone discussions. **Who**: Arabic (n = 1) natural helper with musculoskeletal pain who had undergone a total knee arthroplasty with the service 12-months prior. **How**: The Natural Helper participated in the pre-operative group education session sharing experiences preparing for and undergoing knee arthroplasty surgery, complemented with a minimum of 3 additional points of contact (phone before surgery); virtual visit while the patient was an inpatient; phone or virtual visit as an outpatient in the early post-operative period (3–5 sessions).		

*Mode was necessitated by the Covid-19 pandemic and restrictions on consumer/volunteer and patient activity at each public hospital

CSQ-8: Client Satisfaction Questionnaire-8 item, PAM: Patient Activation Measure, HLQ: Health Literacy Questionnaire

### Outcome measures

A trained outcome assessor collated all outcome measures according to standardised instructions at baseline (treatment commencement) and 3-months post-treatment commencement. It was not possible to blind the outcome assessor due to the sequential phasing of the study (pre-and post-implementation).

Primary feasibility outcome measures and thresholds for progression with a fully powered trial are displayed in Table 2. Process measures of recruitment consenting rates, obtained from clinic recruitment logs, and study retention were collated throughout each study phase. Acceptability was inferred from patient interviews (described below), attendance and satisfaction data. Attendance was collated from therapist logs and calculated as the percentage of the sessions attended, relative to the number of sessions scheduled. Patient satisfaction, evaluated at 3-months post-treatment commencement was assessed using the Client Satisfaction Questionnaire (CSQ-8) [35]. The CSQ-8 is an 8-item questionnaire, translated into Vietnamese and Arabic, with demonstrated reliability and excellent internal consistency for evaluating satisfaction with health services [35]. Respondents are asked to evaluate their experience with treatment using a four-point Likert scale, summated to yield a satisfaction score from 8 to 32 [35]. Conversion of raw scores to a percentage will be used to classify participants as satisfied (CSQ-8 percentage > 50%) or highly satisfied (CSQ-8 percentage > 75%).

**Table 2:** Feasibility Thresholds and Results

Indicator	Threshold	Result P1:P2
**Recruitment**		
Consent rate, %	≥ 50	66:61
Comparability of baseline samples	*p < 0.05*	*p < 0.05*
**Acceptability of treatment**		
Attendance at scheduled appointments, %	≥ 75	75: 89
Participation in mentoring sessions, n	≥ 3	NA: 6
% Participants satisfied with treatment	≥ 75	97:96
**Complete outcome measures, %**	≥ 85	93: 93

P1: Phase one, P2: Phase two, NA: Not Applicable

For clinical outcome measures, a change in activation from baseline to 3-months post-treatment commencement was evaluated for participants in both phases using the Patient Activation Measure (PAM) [36]. The PAM is a reliable and valid 13-item self-reported questionnaire that assesses a patient’s knowledge of their health condition and confidence in managing health-related tasks. Participants are asked to rate their level of agreement on a 5-point scale (0: ‘not applicable’, 1:‘strongly disagree’ through to 4: ‘strongly agree’). Collation of responses across the 13-items is transformed using the developer’s algorithm to a 0–100 metric (0 = lowest activation; 100 = highest activation) and classification of activation level from one (not believing activation is important: ≤47.0) to four (taking action but requiring support to maintain behaviour, ≥67.1). The PAM has been translated into 22 languages and English, Arabic and Vietnamese translation were used in this study.

Additional secondary clinical outcome measures comprised baseline health literacy, assessed using English, Arabic and Vietnamese versions of the Health Literacy Questionnaire (HLQ) [37]. The HLQ is a reliable and valid measure of health literacy, validated for use with socially and culturally diverse patient populations [38]. It consists of 44 items across nine independent domains of health literacy. Five agreement items (graded on a four-point Likert scale) and four capability items (graded on a five-point Likert scale) appraise a respondent’s experiences attempting to understand, access, and use health information and health services [37]. Two items of the HLQ (item 1: feeling understood; item 6: the ability to engage healthcare providers) were also evaluated at 3-months post-treatment to capture trends of change associated with the intervention and/or the utility of the measure as an outcome in future research.

The content of mentoring sessions was evaluated via three methods: mentor debriefing sessions, clinic service records and qualitatively. Data of the number of mentoring sessions for each participant and format were extracted to form a descriptive summary for each case. Across both phases, a purposive sample of participants was invited to participate in a semi-structured individual interview exploring their relationship with healthcare providers, perceived effectiveness of treatment, acceptability of study processes and for the post-phase cohort, their experiences receiving mentoring. Interviews were conducted in a private setting within participating clinics, or via telehealth (according to pandemic restrictions) and overseen and/or conducted by the first author, a female physiotherapist with expertise in musculoskeletal pain and experience in qualitative research methodology. For participants who preferred to speak a language other than English, a female bilingual research assistant, trained in qualitative interviewing and supported by the primary investigator, conducted the interviews directly ‘in language’. The interviewers did not have a prior relationship with any participants. A semi-structured topic guide, developed for this study and piloted with a sample of volunteer community members was used initially and evolved as analysis proceeded alongside data collection [39] (Supplementary file 1). Interviews were digitally audio-recorded, translated (where required) and transcribed for analysis in English. Facilitator reflections were documented immediately following each interview to record contextual information and contribute to reflexivity.

### Sample size and data analysis

While recommendations for sample size in pilot and feasibility studies are varied, a minimum of 20 participants per arm is considered important for informing further research [40]. To allow for the potential for participant drop-out and sufficient numbers for qualitative analysis, we sought to ensure a minimum sample of 30 participants in each phase, across the three cases. To achieve this minimum, a 3-month recruitment period was initially selected, based on projections provided by each clinic. A maximum sample was not stipulated as it was considered opportunistic for informing potential recruitment rates for further research. While sample size calculations were not performed for between-group statistical comparisons, this minimum sample was considered adequate to detect a large effect (0.8) for between-group comparisons for trends of effectiveness (PAM scores).

Due to the unbalanced participant numbers in each phase and non-normal distribution, participant characteristics and feasibility measures are descriptively presented using the median and interquartile ranges for continuous variables; frequencies and proportions (%) for categorical variables. Analysis was conducted using the Statistical Package for the Social Sciences, Version 27 [41]. Between-group comparisons for baseline comparability and trends of effectiveness were conducted using Mann-Whitney U tests or χ2 tests. Missing data were excluded from the analysis.

All qualitative data were read and analyzed by two members of the research team, using a rapid assessment procedure that combined elements of narrative description and the framework method [42, 43]. This approach was selected over more in-depth qualitative analyses because it provided a method for analyzing and interpreting a large volume of qualitative information in a time-efficient manner to directly inform the next phase of research [42, 43]. The analysis followed six main steps. First, a list of key issues and ‘sensitizing concepts’ (or constructs alerting the researcher to possible lines of enquiry) [42] was generated by the research team derived from topics identified before data collection (research questions and topic guide) and key concepts identified during early interviewing (recorded in memos). Subsequently, two researchers (BB and SY) independently read and grouped information from two transcripts according to key issues and sensitizing concepts identified, adding new insights where relevant. The authors compared their provisional analyses and developed a single analytical template to summarize each interview and group information according to key concepts. Successive transcripts were distributed among the research team (BB, SY, RB) and each author read and analyzed successive interviews using the analytical template. The first author (BB) evaluated each analytical template and began constructing a framework matrix in Microsoft Excel. The framework matrix was then used to compare concepts identified in each phase and to understand how experiences varied according to exposure to the mentoring intervention [44]. Key themes were presented as preliminary findings at early meetings with the broader research team, stakeholders and consumers, providing opportunities for discussion, development and refinement. Due to the rapid and iterative nature of this analysis process and the limitations on patient gatherings, imposed by the global pandemic, member checking of transcripts and findings was not performed with individual participants.

Procedures to enhance rigour and trustworthiness were considered throughout the data analysis. Multiple analysts with diverse perspectives were utilized, including two clinical specialist physiotherapists (BB and RB), a Vietnamese multicultural health officer and a bilingual physiotherapy research assistant, who identified with an Arabic-speaking community. Individual interpretations were cross-checked by other members of the research team with previous experience in qualitative analysis to ensure potential bias was considered and findings were scrutinized.

## Results

In phase one, 72 participants were recruited and consented to the study over the extended 6-month recruitment period, of which one participant was not contactable at the 3-month re-assessment (Fig. 1). In phase two, 57 participants were recruited, of which two participants were lost to follow-up at the 3-month outcome assessment (one not contactable and one declined due to unrelated health issues). Recruitment rates were slightly less for the mentoring intervention phase (Table 2). Overall, case three (orthopedics) recruited at a lower rate than cases 1 and 2 for both the pre-implementation and post-implementation cohorts. Analysis of recruitment logs within this case identified previous experience with the intended treatment plan (i.e. a previous joint replacement) as the most differentiating reason for non-participation that was not present in the recruitment logs of other cases. Retention of participants during the study and rates of complete outcome measures were similar between routine care and implementation phases, with both exceeding the threshold of 85% (Table 2). The purposive sample of interviewed participants was 58 (81%) of phase one and 37 (65%) of phase two participants. A larger sample was considered necessary for the pre-implementation phase because a greater heterogeneity of experiences required more participant interviews to achieve sufficient repetition of concepts.

Participant demographics across the two phases were similar (Table 3). Over 80% in both cohorts identified as migrants, spoke a language other than English and identified with a culturally diverse community as their predominant cultural identification. Participants reported comparable living circumstances (although the number of married participants was lower in the post-implementation cohort), educational backgrounds, working status and access to private health insurance. From a symptom perspective, the majority of participants experienced constant symptoms, of long-standing duration and affecting multiple areas of their body. At baseline, patient activation was considered low for both cohorts, with over two-thirds classified as level 1 or 2 activation. Similarly, health literacy across nine dimensions was comparable, with the most marked limitations in having sufficient information to manage health (item 2) and accessing health information (items 8 and 9).

**Table 3:** Participant Baseline Demographic and Symptom Characteristics

	Pre-implementation Cohort (***n*** = 72)	Post implementation cohort (***n*** = 57)
**Age**, median years (IQR)	54.5 (14)	54 (18)
**Gender,** (n) Male: Female	25:47	18:39
**Migrant**, n (%)	58 (81)	49 (86)
Years in Australia, median (IQR)	9.5 (22.5)	7 (19.5)
**Predominant cultural identification,** n (%)		
Australian	13 (18)	8 (14)
Assyrian or Chaldean	23 (32)	23 (40)
Mandaean	12 (16.6)	5 (9)
Arab	10 (13.6)	4 (7)
Vietnamese	5 (7)	5 (9)
Other culturally diverse community	9 (12.5)	12 (21)
**English language proficiency**, n (%)		
Only English	12 (17)	7 (12)
Well or very well	23 (32)	14 (25)
Not well or not at all	37 (51)	36 (63)
**Marital status -** Married n (%)	51 (70.8)	34 (59.7)
**Living Situation**		
With family, n (%)	67 (93.1)	52 (91.2)
**Level of education,** n (%)		
No school or primary	25 (34.7)	21 (36.8)
Secondary	27 (37.5)	22 (38.6)
Tertiary or higher degree	20 (27.8)	14 (24.6)
**Work status**, n (%)		
Full or part-time work	2 (2.8)	5 (8.8)
Unemployed due to pain	51 (70.8)	36 (63.2)
Carer or domestic role	5 (6.9)	8 (14)
Retired	5 (6.9)	3 (5.3)
Other	9 (12.5)	5 (8.8)
**No private health insurance,** n (%)	66 (91.7%)	52 (91.2%)
**Number of comorbidities, median (IQR)**	2 (3)	3 (3)
**Duration of Pain** median (IQR)	10 (8)	10 (11.8)
**Pain symptoms constant**, n (%)	63 (87.5)	50 (87.7)
**Number of areas of pain,** median (IQR)	6 (4)	5 (3)
Classes of medication* /5, median (range)	2 (0–4)	2 (0–4)
**Patient Activation Measure**^**#**^**,** median (IQR)	51 (15.5)	51 (18.4)
**Patient Activation Level**^**#**^ / 4, n (%)		
Level 1	27 (38)	21 (36.8)
Level 2	21 (29)	18 (31.6)
Level 3	18 (25)	14 (24.6)
Level 4	6 (8)	4 (7)
**Health Literacy Questionnaire items**^**#**^		
**Scales of agreement /4,** median (IQR)		
1 Feel understood	3 (0.5)	3 (0.38)
2 Sufficient information	2.75 (0.75)	2.5 (1.0)
3 Actively manage health	3 (0.4)	2.8 (0.8)
4 Social support	2.8 (0.7)	3 (0.7)
5 Appraise health information	2.9 (0.6)	2.8 (0.8)
**Scales of capabilities** /5, median (IQR)		
6 Ability to engage HCP /5	3.6 (1.15)	3.8 (1.1)
7 Navigate health systems /5	3.33 (1.16)	3.5 (1.33)
8 Find good information /5	3.2 (1.2)	3.4 (1.4)
9 Understand health information /5	3.4 (1.0)	3.4 (1.1)

n = Number of participants, % = Percentage within the group, IQR: Interquartile range

^#^Higher value is associated with higher health literacy or higher activation

*Classes of pain medication included simple analgesics, compound analgesics, anti-inflammatory, anticonvulsant, and opioids

*Note: No significant between group differences were observed*

Acceptability of treatment received was inferred from attendance rates, satisfaction, participation in mentoring and qualitative analysis. All feasibility measures met or exceeded those set to define progression to a fully powered trial (Table 2). Median treatment attendance was 75% for the pre-implementation phase and 89% for the post-implementation phase (Table 4). For the post-implementation cohort, participants engaged in a median of six mentoring sessions (range 3–8). In cases one and two, mentoring sessions were conducted weekly, alongside group program appointments, with a median number of 7.5 (range 5–8) and 6 [6] mentoring sessions respectively. For case three, sessions were conducted individually, and the dose was tailored to patient needs. A median of five sessions (range 3–6) was recorded. For participant satisfaction, median CSQ-8 scores were 25 and 27 for the pre- and post-implementation cohorts respectively (Table 4). Similar classifications of satisfaction were observed for both cohorts, with 97% of participants in the pre-implementation phase and 96% in the post-implementation phase satisfied with the treatment. The percentage of participants highly satisfied was higher for the post-implementation cohort (Table 4).

**Table 4:** Treatment Outcomes according to phase

	Pre-implementation cohort	Post-implementation cohort
**Treatment Attendance**	*n = 72*	*n = 56*
Median % attended (IQR)	75 (31)	89 (31)
Number of treatment sessions (range)	6 (0–16)	7 (0–16)
**Patient Satisfaction**	*n = 67*	*n = 53*
CSQ-8 median score /32 (IQR)	25 (8)	27 (6)
Satisfied CSQ > 50%, n (%)	65 (97)	51 (96)
Highly Satisfied, CSQ > 75% n (%)	35 (52)	39 (74)
**Patient Activation**	*n = 71*	*n = 55*
3-month PAM median /100 (IQR)	51 (14.4)	58.1 (25.5)
Change in Activation median /100 (IQR)	0 (11.4)	10.3 (18.9)
3-month PAM level 1 or 2, n (%)	50 (70)	26 (47)
3-month PAM level 3 or 4, n (%)	21 (30)	29 (53)
**Engagement with HCP**	*n = 71*	*n = 55*
3-month HLQ 1 ‘Feeling Understood’ median /4 (IQR)	3 (1.0)	3.25 (1.0)
Change in HLQ 1 median (IQR)	0 (0.5)	0 (0.9)
3-month HLQ 6 ‘Ability to engage HCPs’ median /5 (IQR)	3.8 (1.0)	4 (1.4)
Change in HLQ 6 median (IQR)	0 (0.8)	0 (1.2)

*n* = number of participants, IQR: Interquartile Range, CSQ-8: Client Satisfaction Questionnaire-8 item, PAM: Patient Activation Measure, HCP: Healthcare Provider, HLQ: Health Literacy Questionnaire

Treatment outcomes according to phase are presented in Table 4. The integration of Natural Helper mentoring alongside clinical care (post-implementation) resulted in significantly greater improvement in patient activation throughout treatment than routine care (U = 2643, *p* < 0.01). At 3-months, over half the participants in the post-implementation phase achieved an activation level of three or four, compared with only 30% of the pre-implementation cohort. There was no change in engagement with healthcare providers detected from items one or six of the HLQ over time for either group. The groups were comparable in their self-reported change in pain symptoms across the cases. Routinely collected data for case 1 recorded similar changes in pain severity measured using the Brief Pain Inventory between the pre-implementation (median 0.25, IQR 1.8) and post-implementation cohorts (median 0.5, IQR 1.0). Similarly, self-reported status (same, better, worse) recorded for cases 2 and 3 were comparable with 87% of the pre-implementation cohort reporting they were ‘better’ (13% ‘same’) and 85% of the post-implementation cohort (15% ‘same’).

Qualitatively, three themes characterised the patient experience with treatment and in particular the potential influence a Natural Helper could have on a patient’s treatment journey. These were termed ‘expectational priming’, ‘lived expertise’, and ‘collectivist orientation’. A detailed discussion of each theme and supporting quotes coded as case (C 1–3), participant number (P), cultural identification, and phase (routine care or mentored care) is mentioned below.

### ‘Expectational priming’

Amongst those in routine care, participant expectations rarely deviated from those they held at treatment commencement and these subsequently influenced their depth of treatment engagement. These preconceptions were shaped by the illness model a participant held and the degree to which they considered the treatment approach aligned with this model. For example, if the patient perceived “*physiotherapy cannot treat bone on bone*” (*C2, P60, Assyrian, routine care*), engagement in a physiotherapy exercise-based program was superficial:

“No, it’s not designed for a specific condition, it’s a general treatment. Everyone does it according to his capacity. For example, I suffer from pain in this leg … now, I will see if the doctor will prescribe an operation or not” (C2, P58, Assyrian, routine care).

Amongst those in case two, expectations were framed within a context of a predetermined ‘need’ for surgery, positioning the participant as a passive recipient of care perceiving “I was obliged to do the surgery because I was very sick” (C3, P67, Arab, routine care). In such cases, participant expectations were not oriented towards a self-directed role in the treatment/rehabilitation processes:

“no one helped me before the operation … they just said to have the surgery and they would tell the rest when I am in the hospital” (C3, P69, Chaldean, routine care).

This contributed to a level of disengagement with the rehabilitation process, wherein participants perceived aspects to be “not relevant to my case” (C3, P72, Chaldean, routine care).

For those participants exposed to Natural Helper mentoring, there was a greater reflection on the contribution the mentor had to their treatment expectations, in many cases challenging preconceptions:

“At first, I think it is not suitable for me … I listened to [Natural Helper] and then I find that in the middle of it, its suitable for me” (C1, P92, Vietnamese, mentored care).

Broadened preconceptions and help “on the psychological level..” encouraged many participants to “give it a try and not give up” (C1, P103, Arab, mentored care). They trusted that someone with whom they could identify had mastered these actions. This prompted them to consider change as possible: “when I see her I feel motivated and I think, maybe I can be like her” (C1, P100, Mandaean, mentored care).

### ‘Lived expertise’

A common challenge participants expressed within the routine care cohort was a sense that their experiences living with pain disorders and adopting the recommended behaviours were not well understood by others “who think that I pretend” (C1, P31, Vietnamese, routine care), including family and the broader community,

“my family members, like my wife, didn’t understand my pain … many people didn’t believe in it” (C1, P33, Vietnamese, routine care).

Even among those who appreciated the empathy displayed by healthcare providers, there was an acknowledgement that they couldn’t understand that “my situation is severe” and “there is nothing more you can do for my case” (C1, P45, Arab, routine care).

In the post-implementation phase, the mentor was considered “one of us” (C1, P99, Mandaean, post-implementation), yet at the same time more than another participant attending the program. The Natural Helpers could be trusted to listen to, and empathise with, personal challenges to “remind us about the beauty of life” (C1, P100, Mandaean, mentored care), thereby realizing more holistic dimensions of care that “make me feel as a human” (C1, P105, Arab, mentored care). The lived expertise mentors possessed allowed them to perform roles beyond socioemotional support, including advocating for patients to the clinical team:

“I wanted to say it to [healthcare provider] but I didn’t know how, and she just said it, and I felt so much better because she understood me” (C1, P90, mentored phase).

Mentors’ prior experience with the treatment afforded them a level of expertise analogous to a driving instructor who “knows what he is doing’ because they have “been through this experience before” and therefore can use their mastery “to guide the new driver” (C2, P116, Assyrian, mentored care). In resonating with the mentor’s stories, participants developed optimism that someone had been there before, with all the life complexities of living with chronic conditions and managing personal responsibilities. Even when participants did not experience a change in perceived outcome (“I can’t say my pain is better”, C1, P98, Mandaean, mentored care), they continued to look to the future, communicated enhanced ownership and a sense of personal responsibility for making change happen:

“I understood everything depends on my personal effort” (C1, P97, Chaldean, mentored care).

### Collectivist orientation

Across both cohorts, participants highly valued communal sharing and problem-solving. In all cases, there was an opportunity for communal engagement in the form of a group education session or co-location of rehabilitation (case three) and/or group programs (cases one and two). Participants highly valued this opportunity, with most considering communal engagement as fundamental to their culture:

“It is our culture, we are like 24 hours with the group … everything we share, we didn’t eat anything without your partner, your friend, or sharing it with other people” (C1, P49, Arab, routine care).

For many in the routine care cohort, a missed opportunity was communicated, wherein participants considered “we didn’t get to communicate a lot … if we could sit together for 30 minutes, we could have known each other” (C2, P58, Assyrian, routine care). Participants felt rigid program structures did not prioritise sociocultural interaction, despite the opportunity for group engagement playing a large role in their motivation or desire to attend the sessions (“in a team is more enthusiastic”, C2, P60, Assyrian, routine care). Throughout the mentored phase, social participation amongst those receiving treatment remained limited, especially when pandemic restrictions constrained group interaction. However, the therapeutic benefit of social connectedness was realized via individual time with their respective mentor whose “tenderness and patience” (C2, P119, Chaldean, mentored care) added another dimension to the care provided by the healthcare team; “we do need her, we actually need the whole team, because if one pillar is missing, the whole thing will fall down” (C1, P103, Arab, routine care).

Amongst those in case one, where participants could identify with any ethnocultural orientation, perspectives of the therapeutic value of social interaction and mentoring were mixed. Those who valued collectivism, especially those identifying with Assyrian, Mandaean, Arab and Vietnamese cultures, overwhelmingly endorsed the mentoring initiative and desired to promote the programs amongst their respective communities:

“I think this program needs to be disseminated in the community, the Vietnamese community” (C1, P92, Vietnamese, mentored care) and.

“Despite all the pains that I have, I am ready to support everyone around me” (C1, P100, Mandaean, mentored care).

Amongst those who identified more strongly with individualistic values, in particular those who were identified predominantly with Australian culture, weaker therapeutic benefits were reported. These participants were less likely to perceive the relationship with other participants or mentors as beneficial beyond an initial normalization of the pain experience.

“at the beginning, I think they [Natural Helper] can give you extra information about the program in case you join with some level of skepticism … but in the future may be less so” (C1, P83, Australian, mentored care).

In such circumstances, the desire was stronger to follow the instruction of the healthcare provider who had expertise, feeling that the mentor’s approach “was not scientific” (C1, P78, Australian, mentored care).

## Discussion

The Natural Helper program was designed to target pain management challenges experienced by vulnerable communities attending different forms of management for musculoskeletal conditions. Results from this pilot study, including the achievement of all thresholds for progression, suggest it is feasible to implement, and subsequently evaluate a mentoring initiative targeted toward specific cultural communities with chronic musculoskeletal conditions. Further, patient reported outcomes suggested there may be an advantage in favour of mentoring, relative to routine care, for improving activation and participation in treatment.

While patient activation has been extensively studied in the chronic disease literature [45], few studies have explored applying it to pain management [46, 47]. In our study, the median activation was 51 with 67% of participants classified ‘low‘(level 1 or 2) activation at baseline. These scores and rates are lower than findings observed in other studies exploring PAM values in adults with persistent pain [46, 48]. A cross-sectional survey of Chinese patients with chronic pain attending a Chinese Medicine Centre revealed mean activation levels of 56.6 (15.4), of which 51.6% of the cohort were classified with low activation [46]. Similarly, an Australian study of English-speaking patients with end-stage osteoarthritis revealed higher mean activation levels (60.5, SD 11.0) and only 26% of their cohort classified with low activation [48]. Sociodemographic factors that are associated with activation levels, such as health literacy, societal language proficiency, socioeconomic status and educational attainment [45, 46, 49] may account for the differences observed between our cohort and these previous studies. Specifically, the intersection between migration status, low English-language proficiency and challenging socioeconomic circumstances (Table 3) may have contributed to an inequitable starting point, as observed in other research [6]. Combined with observations that higher activation levels are associated with improved patient self-management and patient-reported outcomes [50] our findings of low starting activation levels that resist changes with routine care provide a strong rationale for targeted intervention in vulnerable communities.

Empowering patients to adopt self-management behaviours was an important component of treatment across the three cases included in this study. However, this is recognized as more challenging for patients from CALD backgrounds, who are observed to have higher rates of treatment non-attendance, drop out and disengagement with healthcare concepts than the wider population [16]. This may explain why a change in activation with routine care was not achieved, with participants highlighting low expectations that treatment could change their situation (*theme: expectational priming*) and a sense of disconnect from their healthcare providers (*theme: lived expertise*). Our rationale for exploring social aspects of pain management is supported by a recent systematic review of patient experiences of pain management that highlighted receiving emotional and motivational support and having an opportunity to share their concerns with others as key facilitators of treatment success [51]. For CALD communities, this therapeutic effect may be more pronounced among those who align with fundamental cultural values of collectivism, where social cohesiveness and interdependence are prioritized and strongly linked to identity [52]. Indeed, patients who communicated collectivist values in our study perceived stronger therapeutic benefits of social participation and exposure to the Natural Helper mentors (*theme: collectivist orientation*). Thus, there may be a differential effect of the value of social support according to cultural identification. Recent research has suggested there are varied mechanisms via which social support achieves therapeutic benefit [53]. Improvements in stress appraisal, self-efficacy and the adoption of adaptive coping strategies are generally recognized, however, the authors concluded that different types and contexts of social support may be more efficacious than others [53], warranting further exploration.

Several potential mechanisms explain the therapeutic benefit associated with mentoring observed in our study. Self-efficacy, defined as an individual’s belief in their ability to successfully perform a behaviour necessary to produce a specific outcome [54], is a component of patient activation that is significantly correlated with patient empowerment and self-management [50, 55]. While pain self-efficacy was not directly measured in this study due to the lack of reliable and valid language translations, analysis of participant accounts provided some insight into self-efficacy as a mechanism for change. Mentors’ prior success utilizing treatment components acted as a form of vicarious experience, an antecedent of self-efficacy [56]. Social cognitive theory suggests the more relatable the person modelling the successful behaviour, the greater an individual will perceive their success with the same actions [56]. Certainly, this was evident across themes of ‘*expectational priming’ and ‘lived expertise’*, where participants identified with the mentors and were motivated by their outcomes. Not only did participants acknowledge achieving the desired outcome was possible, but they began to consider their actions as directly related to an outcome. Such belief is considered a prerequisite for the successful adoption of behaviours necessary for long-term self-management and improved health outcomes [45]. Further research is needed to understand if the observed improvements in patient attendance and activation correspond to improvements in condition-specific outcomes, as has been observed for other chronic diseases [45].

While this study contributes knowledge informing the potential value of culture-specific peer mentoring for people with musculoskeletal pain, limitations must be considered. The cases selected for inclusion were based on convenience and only included hospital-based clinics in a single local health district in Australia. Further, 73% of participants included were recruited from one case, a tertiary pain clinic. As such, the experiences may not be representative of the broader community living with chronic musculoskeletal disorders or those who manage musculoskeletal disorders in primary care. Second, the study was designed to pilot the intervention and as such was not powered for clinical effectiveness outcomes. The clinical outcomes presented are therefore exploratory and cannot infer about the effectiveness of cultural mentoring beyond the current sample. Nonetheless, this cohort provided the opportunity to explore the feasibility of the intervention and evaluation processes, such as evaluating the utility of some outcome measures (HLQ items). Third, the choice of a Rapid Assessment Approach for the qualitative analysis may have affected the depth of the thematic analysis and subsequent understanding of the patient experience. Nonetheless, research suggests when applied with rigour, this approach can produce overlapping results compared with traditional thematic analysis, while also reducing the time and cost of in-depth qualitative analyses [57, 58]. Last, the availability of reliable and valid translated tools and the known low health literacy of target populations constrained the number and choice of outcome measures selected to evaluate trends of effectiveness or potentially confounding variables, such as pain medication regimens. Therefore, the extent to which changes in activation are attributable to pain-specific outcomes or other variables is unknown. Further research would benefit from including health outcomes that link mechanisms of action (such as activation) with relevant long-term outcomes.

## Conclusions

Implementing a peer mentor model of care alongside routine care across three musculoskeletal pain settings was feasible and acceptable to patients from diverse cultural backgrounds. Culture-specific mentors appeared to be instrumental in empowering patients to adopt the self-management behaviours considered important for effective pain management. The findings of improved patient activation, satisfaction and attendance may have implications for other chronic disease settings where active self-management is a critical element.

## Supplementary Information

**Additional file 1.**

**Additional file 2.**

**Additional file 3.**

## Data Availability

Reasonable requests for data are available by contacting the corresponding author at Bernadette.brady@health.nsw.gov.au
