# Supplementary material for: The feasibility of implementing a cultural mentoring program alongside pain management and physical rehabilitation for chronic musculoskeletal conditions: results of a controlled before-and-after pilot study
Source: BMC Musculoskelet Disord. 2023 Jan 19;24:47. doi: 10.1186/s12891-022-06122-x (PMC9850562; doi:10.1186/s12891-022-06122-x)
Supplement: Supplementary file 1 — Additional file 1. [file 12891_2022_6122_MOESM1_ESM.pdf]

Manuscript: The feasibility of implementing a cultural mentoring program alongside pain management and physical rehabilitation: Results of a controlled before-and-after feasibility study.

Interview Guide

Aim	Proposed questions
**Overall impressions of treatment**<br><br>To understand how applicable patients felt the treatment and its components were for their situation	**1. You have recently attended the [*insert clinic name*] for treatment of your [*insert condition*]. How would you describe your overall experience with treatment?**<br><br>*[Prompts: Were there things you found helpful or unhelpful about your treatment?]*<br><br>***2.*** **As you know the [*insert treatment or program name*] is designed to cater for people with different pain experiences. How applicable do you think the program/treatment was to your situation?**<br><br>*[Prompts: In what ways (if any) did it address your specific problems? How did you feel you compared to other people in the program (if applicable); How closely was the program tailored to your needs?]*
**Level of support**<br><br>To understand how supported patients felt during their treatment period	**3. Overall, what were the things that made it easier or harder for you to attend or participate in your treatment?**<br><br>*[Prompt for specific domains if needed: For example, what role did the following play, if any:*<br>*a) social or community network (ie. other participants (if applicable), family or community);*<br>*b) healthcare providers*<br>*c) other characteristics such as transport, cost, and time]*<br><br>**4. In your opinion could the [*insert clinic name*] have done anything differently to make it easier for you to**<br>**a) attend or participate or apply in your treatment/program?**<br>**b) put the exercise, education, or treatment strategies into practice?** (*please elaborate)*<br><br>**5. Thinking about your relationship with the healthcare team who were involved in your treatment. How would you describe your interaction or experience with them?**<br><br>*[Prompts: What did you find helpful or unhelpful about them/ their style / their practice? Is there anything they could have done to support you better?]*<br><br>**6. Thinking about the other patients you may have interacted with as part of your treatment. How would you describe your interaction or experience with them?**<br>*[Prompts: How would you describe your relationship with them? Is there anything they could have done to support you better?*

**Cultural and social responsiveness of treatment**<br><br>To understand how aligned the program and its content were to the patient's cultural and social values	**7. Some people feel their cultural background, such as belonging to [insert culture] influences their pain or how applicable a treatment program may be to them. Based on your experience, how applicable do you feel the program/treatment was for you?**<br><br>*[Prompts: Explore if needed the influence of cultural identification between patients and therapists; patients and other participants; expectations for treatment].*<br><br>**8. Some people feel their personal, family or other responsibilities influence their ability to attend or participate in treatment. What influence, if any did your responsibilities have on your experience with treatment?**<br><br>*Prompts: What, if anything, could have been done to support you better with these things?*
**Intervention Cohort Specific Questions**	**9. I am interested to know your thoughts on the mentor that you interacted with as part of your treatment [insert mentor name].**<br>**- How did you feel about [insert name] interacting with you as part of your treatment?**<br>**- What, if anything, was helpful or unhelpful about interacting with this person?**<br><br>**10. During your interaction with [insert mentor name], were there any concerns or challenges you encountered?**<br><br>**11. If you were to do the program/treatment again and had the option of choosing between a mentor or no mentor for your treatment. Which would you choose based on your experience, and why?**
**Recommendations** (*If not already canvassed)*	**12. In what ways, if any, could your treatment/program have been improved?**<br><br>**13. If a family member or someone from your community came to you with a similar condition and asked about your experience with [insert clinic name]. Would you recommend they attend? Why and why not?**
**Concluding**	**14. Overall, how satisfied are you with the program you attended?**<br><br>[*Prompts: How could the health system/[insert clinic] help you better?*<br><br>This concludes our main questions. Is there anything else we have not discussed that you would like to raise with me about your experience with the treatment program offered by… Thank you.
