# Supplementary material for: The feasibility of implementing a cultural mentoring program alongside pain management and physical rehabilitation for chronic musculoskeletal conditions: results of a controlled before-and-after pilot study
Source: BMC Musculoskelet Disord. 2023 Jan 19;24:47. doi: 10.1186/s12891-022-06122-x (PMC9850562; doi:10.1186/s12891-022-06122-x)
Supplement: Supplementary file 3 — Additional file 3. [file 12891_2022_6122_MOESM3_ESM.pdf]

Manuscript: The feasibility of implementing a cultural mentoring program alongside pain management and physical rehabilitation: Results of a controlled before-and-after feasibility study.

Consolidated criteria for reporting qualitative studies (COREQ): 32-item checklist

Developed from:

Tong A, Sainsbury P, Craig J. Consolidated criteria for reporting qualitative research (COREQ): a 32-item checklist for interviews and focus groups. *International Journal for Quality in Health Care*. 2007. Volume 19, Number 6: pp. 349 – 357

No. Item	Guide questions/description	Reported on Page #
**Domain 1: Research team and reflexivity**		
Personal Characteristics		
1. Inter viewer/facilitator	Which author/s conducted the inter view or focus group?	Page 13
2. Credentials	What were the researcher's credentials? E.g. PhD, MD	Page 13
3. Occupation	What was their occupation at the time of the study?	Page 13
4. Gender	Was the researcher male or female?	Page 13
5. Experience and training	What experience or training did the researcher have?	Page 13
Relationship with participants		
6. Relationship established	Was a relationship established prior to study commencement?	Page 13 .
7. Participant knowledge of the interviewer	What did the participants know about the researcher? e.g. personal goals, reasons for doing the research	Page 13
8. Interviewer characteristics	What characteristics were reported about the inter viewer/facilitator? e.g. Bias, assumptions, reasons and interests in the research topic	Page 14

**Domain 2: study design**		
Theoretical framework		
9. Methodological orientation and Theory	What methodological orientation was stated to underpin the study? e.g. grounded theory, discourse analysis, ethnography, phenomenology, content analysis	14
Participant selection		
10. Sampling	How were participants selected? e.g. purposive, convenience, consecutive, snowball	Page 13
11. Method of approach	How were participants approached? e.g. face-to-face, telephone, mail, email	Page 8, 9
12. Sample size	How many participants were in the study?	Page 13-14
13. Non-participation	How many people refused to participate or dropped out? Reasons?	Page 13-14
Setting		
14. Setting of data collection	Where was the data collected? e.g. home, clinic, workplace	Page 13 .
15. Presence of non-participants	Was anyone else present besides the participants and researchers?	Page 13 Inferred as one to one interviews
16. Description of sample	What are the important characteristics of the sample? e.g. demographic data, date	Table 3
Data collection		
17. Interview guide	Were questions, prompts, guides provided by the authors? Was it pilot tested?	Supplementary file 1, 13
18. Repeat interviews	Were repeat inter views carried out? If yes, how many?	No, inferred on page 13
19. Audio/visual recording	Did the research use audio or visual recording to collect the data?	Page 13,

20. Field notes	Were field notes made during and/or after the interview or focus group?	Page 13
21. Duration	What was the duration of the interviews or focus group?	
22. Data saturation	Was data saturation discussed?	Page 17
23. Transcripts returned	Were transcripts returned to participants for comment and/or correction?	Page 15
**Domain 3: analysis and findings**		
Data analysis		
24. Number of data coders	How many data coders coded the data?	Page 14-15
25. Description of the coding tree	Did authors provide a description of the coding tree?	No
26. Derivation of themes	Were themes identified in advance or derived from the data?	Page 14-15
27. Software	What software, if applicable, was used to manage the data?	Page 14-15
28. Participant checking	Did participants provide feedback on the findings?	Page 15
Reporting		
29. Quotations presented	Were participant quotations presented to illustrate the themes/findings? Was each quotation identified? e.g. participant number	Page 18-22
30. Data and findings consistent	Was there consistency between the data presented and the findings?	Yes, there was. Page 18-22
31. Clarity of major themes	Were major themes clearly presented in the findings?	Yes. they were. From 18-22
32. Clarity of minor themes	Is there a description of diverse cases or discussion of minor themes?	Discussion of major and minor themes From 18-22
